# Reconcile the debate over protective effects of BCG vaccine against COVID-19

**DOI:** 10.1038/s41598-021-87731-9

**Published:** 2021-04-16

**Authors:** Wei Fu, Pei-Chuan Ho, Chia-Lun Liu, Kai-Teh Tzeng, Nawar Nayeem, Jonni S. Moore, Li-San Wang, Shin-Yi Chou

**Affiliations:** 1 Penn Neurodegeneration Genomics Center, Perelman School of Medicine, University of Pennsylvania, Richards Building, D101, 3700 Hamilton Walk, Philadelphia, PA 19104 USA; 2 Department of Pathology and Laboratory Medicine, Perelman School of Medicine, University of Pennsylvania, Philadelphia, PA 19104 USA; 3 Department of Economics, Lehigh University, Rauch Business Center, 621 Taylor Street, Bethlehem, PA 18015 USA; 4 National Bureau of Economic Research, Cambridge, MA USA

**Keywords:** Viral infection, Health policy, Epidemiology

## Abstract

While awaiting the COVID-19 vaccines, researchers have been actively exploring the effectiveness of existing vaccines against the new virus, among which the BCG vaccine (Bacillus Calmette-Guérin) receives the most attention. While many reports suggest a potential role for BCG immunization in ameliorating SARS-CoV-2 infection, these findings remain controversial. With country-level COVID-19 outbreak data from Johns Hopkins University Coronavirus Resource Center, and BCG program data from World Atlas of BCG Policies and Practices and WHO/UNICE, we estimated a dynamic model to investigate the effect of BCG vaccination across time during the pandemic. Our results reconcile these varying reports regarding protection by BCG against COVID-19 in a variety of clinical scenarios and model specifications. We observe a notable protective effect of the BCG vaccine during the early stage of the pandemic. However, we do not see any strong evidence for protection during the later stages. We also see that a higher proportion of vaccinated young population may confer some level of communal protection against the virus in the early pandemic period, even when the proportion of vaccination in the older population is low. Our results highlight that while BCG may offer some protection against COVID-19, we should be cautious in interpreting the estimated effectiveness as it may vary over time and depend on the age structure of the vaccinated population.

## Introduction

The first confirmed case of COVID-19 (Coronavirus Disease 2019) was reported on January 2, 2020 in Wuhan, a metropolitan area in central China, and the causative agent of this outbreak was identified as a new strain of coronavirus, designated SARS-CoV-2. As of November 11, 2020, there are more than 50 million confirmed cases, 1.2 M deaths, and the numbers continue to climb. COVID-19, the disease caused by SARS-CoV-19, is highly infectious, with an R0 estimate between 0.4 and 4.6^1^. COVID-19 has a substantially higher morbidity rate and mortality rate for people 60 years and above^2,3^ and for people with chronic conditions including pulmonary disease, hypertension, diabetes mellitus, and cardiovascular disease^4^. Treatment options are limited although there has been recent progress in both therapeutic options and vaccine development recently^5–7^.

As targeted COVID-19 vaccines are being developed, researchers have been actively exploring the protective effects of existing vaccines, among which the BCG vaccine (Bacillus Calmette-Guérin) has received much attention following observation of a coincident relationship between severity of COVID-19 outbreaks and the level of BCG vaccination in one country^8^. The bacille Calmette-Guérin (BCG) vaccine was introduced in the 1930s and used to prevent tuberculosis (TB), which is one of the most prevalent diseases in developing countries^9,10^. In the 1970s, WHO launched massive immunization programs in developing countries, leading to substantial increase in BCG coverage among 1-year-olds: BCG coverage rate increased from 9% to 80% in African countries, and from 20% to 90% in European countries between 1980 and 2019^11^. Though BCG vaccine was first developed as a prevention for tuberculosis, recent studies have found that BCG vaccine has nonspecific benefits in training the innate immunity through bestowing a type of immunological memory on innate cells^12–14^. This “trained innate immunity” has been shown to be protective against viral infections^15,16^.

The protective effect of BCG vaccination against COVID-19 is under debate^17–20^. While the negative correlation between BCG vaccination and mortality and morbidity rates is observed^19,21–23^, studies like Hamiel et al.^17^ and Lindestam Arlehamn et al.^20^ reported no significant protection of BCG vaccination. Escobar et al.^19^ compared the COVID-19 death records in the early stages of the pandemic (up to April 22, 2020) from countries with a national BCG vaccination program to countries without, and observed significant BCG protective effect on COVID-19 mortality. Yet other studies found no significant correlation between COVID-19 mortality and BCG vaccination. For example, Lindestam Arlehamn et al.^20^ failed to validate the protectiveness of BCG using updated data from August 2020, and Patella et al.^24^ do not support significant differences in the COVID-19 incidence between “BCG vaccinated” and “BCG unvaccinated” physicians in Italy. This study aims to explain the conflicting findings from existing studies by examining the impact of BCG vaccination during different stages of pandemic.

We construct a dynamic model to investigate the effect of BCG vaccination, allowing its protection to vary across time during the pandemic. Our results reconcile the existing different opinions towards the protection of BCG against COVID-19 robustly in a variety of samples and model specifications. Our first explanation for the discrepancy between the controversial findings is that protectiveness of BCG varies over time during the pandemic. We observe a notable reduction in COVID-19 mortality in countries with a national BCG vaccination program, compared to countries without such a program during the second and the third month since the first death confirmed. This echoes Escobar et al.^19^ in that the protective effect of BCG vaccine, at least to some extent, existed during the early stage of the pandemic. However, we do not see strong evidence for protectiveness during the later stages, which is broadly in line with Lindestam Arlehamn et al.^20^. Our second explanation is that the age structure of the BCG “vaccinated” population matters. We see that a higher proportion of vaccinated young population helps to protect the society against the virus, while the proportion of vaccinated old population seems to show limited effects.

## Methods

### COVID-19 data

We obtain country-level COVID-19 outbreak data from Johns Hopkins University Coronavirus Resource Center^25^. The dataset includes the daily numbers of confirmed COVID-19 cases and the numbers of COVID-19 deaths reported by 188 countries between January 22, 2020 and October 15, 2020. Since the timing of the outbreak differs from country to country, we construct a dataset that tracks each country from the day before the first confirmed death and up to 210 days after the “onset.”

### BCG vaccination program by country

Two types of data are considered. First, World Atlas of BCG Policies and Practices^26^ provides country-level beginning and ending years for a national BCG vaccination program; we reviewed literature and governmental websites to fill in missing information whenever possible. Second, WHO/UNICEF provides Estimates of National Immunization Coverage (WUENIC) monitoring the coverage of fourteen vaccines during 1990–2018 (latest update July 1 2019^11^). See Supplementary Table 1 (with references) and Supplementary Figs. 1–4.

### Government response index

Government response data are obtained from Hale et al.^27^, which contain publicly available information about government responses coded into eight indicators for containment and closure, one indicator for health system (“public info campaigns”), two economic indicators (“economic support” and “debt/contract relief for households) and three indicators for health measures (“testing policy”, “contact tracing” and “facial coverings”). Note that one country’s public health system does matter in controlling the pandemic and may also influence the implementation of the BCG vaccination program, we control for the government response index in our model to capture the time-varying confounders in the public health system. An alternative index available in Hale et al.^27^ is the policy stringency index. We prefer the government response index to the policy stringency index because the latter does not take health measures such as testing policy and facial coverings into account. We present our main results using the government response index and also use the policy stringency index which counts eight containment policy indicators and the indicator for health system for robustness check.

### Quality control of data

A country is excluded from our analysis if it has 0 confirmed cumulative death as of the last day in our analysis (October 15, 2020) and has missing value in the government response index data. See Supplementary Tables 3–5 for countries that we included in the main analysis.

### Regression analysis

Three regression models were employed to estimate the impact of BCG vaccination while controlling for possible confounding factors. To mitigate confounding effects, we incorporate the government response index to capture the governmental efforts, country fixed effect which helps to control for time-invariant country characteristics, and day fixed effect (since the first confirmed death) to absorb the potential confounders along with the timing of outbreak.

In comparing across countries, we define day 1 for each country as the day the first confirmed death is reported and day 0 for the last day prior to the death reported. This is similar to the approach in Escobar et al.^19^, which compares the COVID-19 mortality data at specific times of each country epidemic since the first death. Adjusting time according to the first day of death allows us to “align” the timelines of epidemics across countries and examine the mortality data across countries that are in the same pandemic stage. Our three models estimate the impact of BCG vaccinations per day (starting from day 31) with reference to the first month after the first death (day 0 to day 30).

1. Model 1 estimates how current and past national BCG vaccinations affect the number of cumulative deaths per day at country level:
  1 $$\begin{aligned} ln\left( {Y_{ij} } \right) & = \mathop \sum \limits_{k = 31}^{K} \alpha_{k} \,AllBCG_{i} \times day_{k} + \mathop \sum \limits_{k = 31}^{K} \beta_{k} \,HadBCG_{i} \times day_{k} \\ & \quad + Gov^{\prime}t \,Response_{ij} + CountryFE + DayFE + \varepsilon_{ij} , \\ \end{aligned}$$
  where $Y_{ij}$ is the primary outcome, the cumulative number of deaths, for country $i$ at day $j$($0 \le j \le 210)$. Day 0 is denoted as the day before the first confirmed death. We set BCG program indicators $AllBCG_{i} = 1$ or $HadBCG_{i} = 1$ if the country $i$ has a national BCG vaccination program currently or in the past respectively. The reference group is the countries without the BCG vaccination program. We take the first month after the first death reported as the reference period. The coefficient ($\alpha_{k}$) of $AllBCG_{i} \times day_{k}$ is informative of the protective effect across all countries with ongoing national BCG vaccination programs in each day $k$ after the first month.
2. In the 1970s, WHO launched massive immunization programs, including the BCG vaccination, in developing countries. The BCG vaccination was administered on newborns and young children. As a result, people under 50 years old in countries that have undergone the massive immunization program have a much higher vaccination rate than those 50 years and older. Model 2 estimates the impact of BCG coverage in younger and older populations in the country.
  2 $$\begin{aligned} ln\left( {Y_{ij} } \right) & = \mathop \sum \limits_{k = 31}^{K} \alpha_{k} Under50_{i} \times day_{k} + \mathop \sum \limits_{k = 31}^{K} \beta_{k} \,Over50_{i} \times day_{k} \\ & \quad + Gov^{\prime}t \,Response_{ij} + CountryFE + DayFE + \varepsilon_{ij} , \\ \end{aligned}$$
  where $Under50_{i}$ is the proportion of years between 1970 and 2019 with a national BCG vaccination program in country $i$. This variable captures the BCG coverage of the population under age of 50. The variable $Over50_{i}$ is defined similarly as the proportion of years between 1935 and 1969 to capture the BCG coverage of population between ages 51 and 85. As robustness checks, we also re-analyze model 2 with different age cut-offs (e.g., 40-, 45-, and 55-year-old) and results are consistent (see Supplementary Figs. 6–8).
3. Model 3 estimates the impact of BCG vaccination as an average coverage between 1990 and 2018. As shown in Supplementary Fig. 5, there is a clear gap in the density around the first quintile (coverage: 82.84%) of the average BCG vaccination coverage, which explicitly splits the countries into two groups. Model 3 compares the COVID-19 related deaths between countries with high BCG coverage and countries with low BCG coverage.
  3 $$\begin{aligned} ln\left( {Y_{ij} } \right) & = \mathop \sum \limits_{k = 31}^{K} \alpha_{k} BCGHighCoverage_{i} \times day_{k} \\ & + Gov^{\prime}t\, Response_{ij} + CountryFE + DayFE + \varepsilon_{ij} , \\ \end{aligned}$$
  where $BCGHighCoverage_{i}$ = 1 if the average coverage of 1-year-olds receiving one dose of BCG vaccine between 1990 and 2018 in country $i$ is higher than the first quintile (2nd quintile and above) across all countries, and otherwise 0. Greece is excluded since it has less than 10 years of record with BCG coverage data. We also perform analysis leveraging a continuous BCG coverage metric in Supplementary Table 8.

All three models include fixed effect terms by country (vector $CountryFE$) and day (vector $DayFE$). Outcome variables are cumulative confirmed deaths per day after log transformation ($ln(Y_{ij} )$). The cumulative deaths are transformed by log(deaths + 0.0001) since log of 0 is not defined. Standard errors are clustered at country level.

After presenting the dynamic effects of BCG using Models 1–3, we summarize the effects of BCG vaccination in a reduced version. Specifically, taking Model 1 as an example, we estimate the following equation:

4 $$\begin{aligned} ln\left( {Y_{ij} } \right) & = \mathop \sum \limits_{k = 1}^{3} \alpha_{k} AllBCG_{i} \times Period_{k} + \mathop \sum \limits_{k = 1}^{3} \beta_{k} HadBCG_{i} \times Period_{ k} \\ & \quad + Gov^{\prime}t \,Response_{ij} + CountryFE + DayFE + \varepsilon_{ij} , \\ \end{aligned}$$

where we group $day_{{}}$ into four periods: days 0–30, days 31–90, days 91–150, and days 151 and beyond. This revised model estimates the average effects of BCG for different periods after the first month of pandemic onset. Note that the summation only has three periods since the first period is the reference period.

In order to explore the heterogeneous effects of BCG vaccination along with government policy against COVID-19 in a country, we further extend Eq. (4) by incorporating full interaction terms between BCG measures, time periods, and government response index as follows:

5 $$\begin{aligned} ln\left( {Y_{ij} } \right) & = \mathop \sum \limits_{k = 1}^{3} \alpha_{k} AllBCG_{i} \times Period_{k} + \mathop \sum \limits_{k = 1}^{3} \beta_{k} HadBCG_{i} \times Period_{k} \\ & \quad + Gov^{\prime}t \,Response_{ij} + CountryFE + DayFE \\ & \quad + \eta_{1} AllBCG_{i} \times Gov^{\prime}t \,Response_{ij} + \eta_{2} HadBCG_{i} \times Gov^{\prime}t \,Response_{ij} \\ & \quad + \mathop \sum \limits_{ k = 1}^{3} \rho_{k} Period_{k} \times Gov^{\prime}t \,Response_{ij} \\ & \quad + \mathop \sum \limits_{k = 1}^{3} \lambda_{k} AllBCG_{i} \times Period_{k} \times Gov^{\prime}t \,Response_{ij} \\ & \quad + \mathop \sum \limits_{k = 1}^{3} \theta_{k} HadBCG_{i} \times Period_{k} \times Gov^{\prime}t \,Response_{ij} + \varepsilon_{ij} , \\ \end{aligned}$$

where $\lambda_{k}$ and $\theta_{k}$ capture the heterogeneous protectiveness of BCG vaccination under different isolation policy. For example, $\lambda_{k} > 0$ implies that the protective effect of BCG vaccination would be weaker in reducing mortality as increasingly stringent isolation policy is implemented. We also extend model 2 in a similar way by replacing $AllBCG_{i}$ and $HadBCG_{i}$ with $Under50_{i}$ and $Over50_{i}$, and extend model 3 by interacting with $BCGHighCoverage_{i}$.

### Robustness check

We rerun our regression models with the following different sample restrictions (per model) to check the robustness of our results. First, a country is excluded if its population is less than 1 million^8,21,22,28,29^. Second, countries are divided into high, upper-middle, lower-middle, and low income groups based on World Bank Gross National Income per capita data^30^. Countries in the low income group are excluded^8^. For the details of each sample, please see Supplementary Table 2. We also report results using policy stringency index in place of the government response index. Besides, we replicate all our results in countries with a human development index no less than 0.7^19^.

## Results

**Figure 1:**
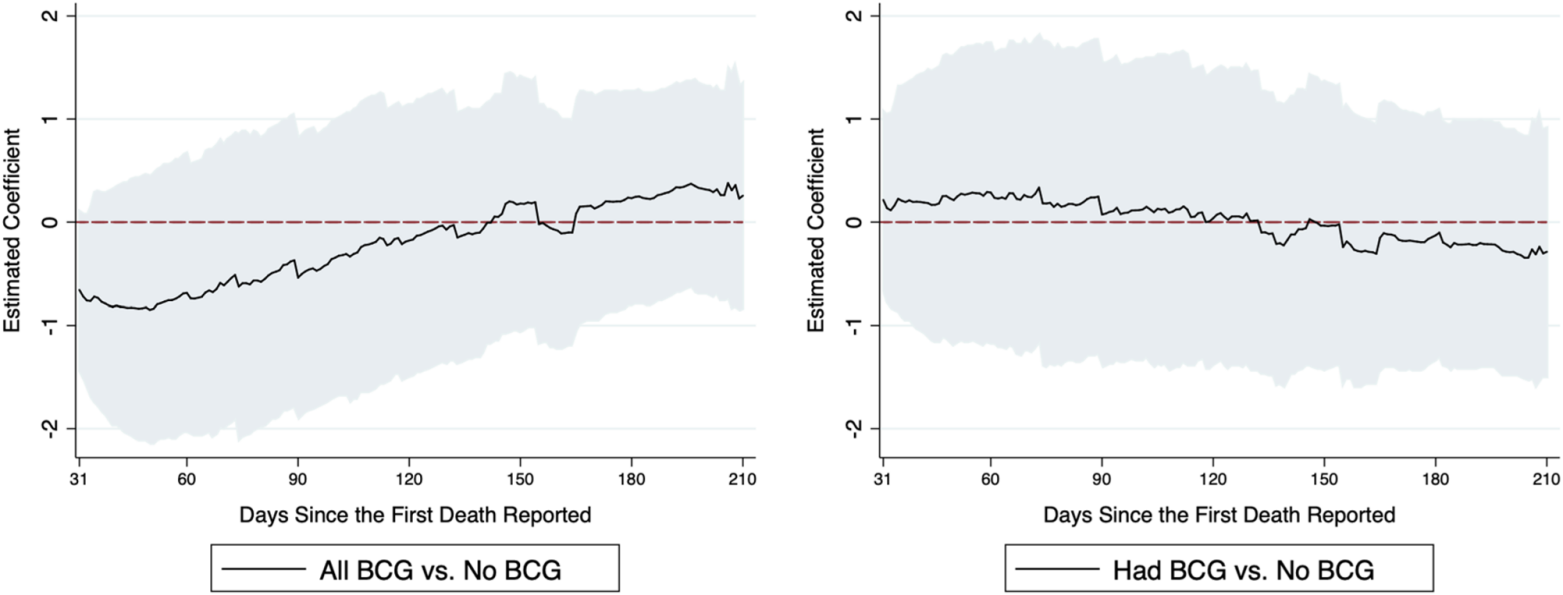
(Model 1): This figure summarizes the estimated effects of all-BCG and had-BCG on cumulative deaths in logarithm since the first death reported. The coefficients are estimated effects of BCG vaccination program for day 31 to day 210, compared to the first 30 days. On the left of the figure are the estimates of $\alpha_{k}$ in Eq. (1), and on the right are the estimates of $\beta_{k}$ in Eq. (1).

**Figure 2:**
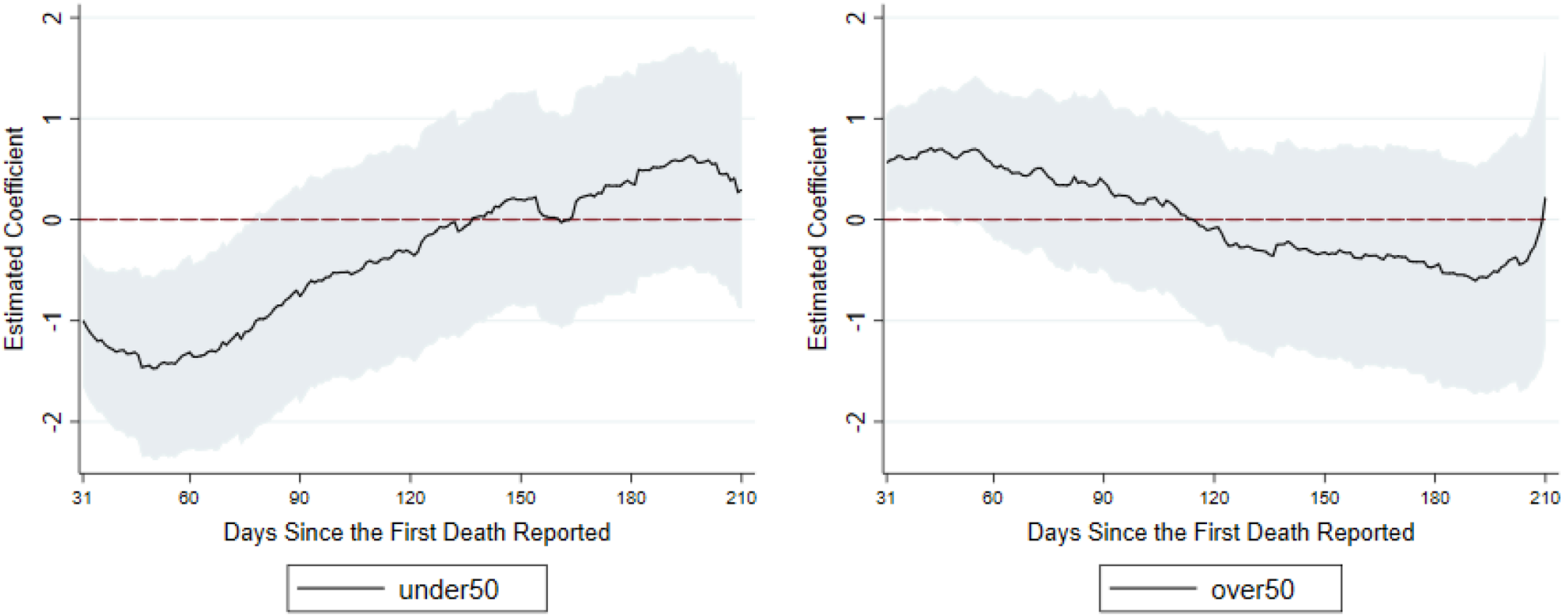
(Model 2) This figure summarizes the estimated effects of under50 and over50 on cumulative deaths in logarithm since the first death reported. The coefficients are estimated effects of BCG vaccination program for day 31 to day 210, compared to the first 30 days. On the left are the estimated under50 effects on weekly log(deaths) which are estimates of $\alpha_{k}$ in Eq. (2). On the right are the estimated over50 effects on weekly log(deaths) which are the estimates of $\beta_{k}$ in Eq. (2).

**Figure 3:**
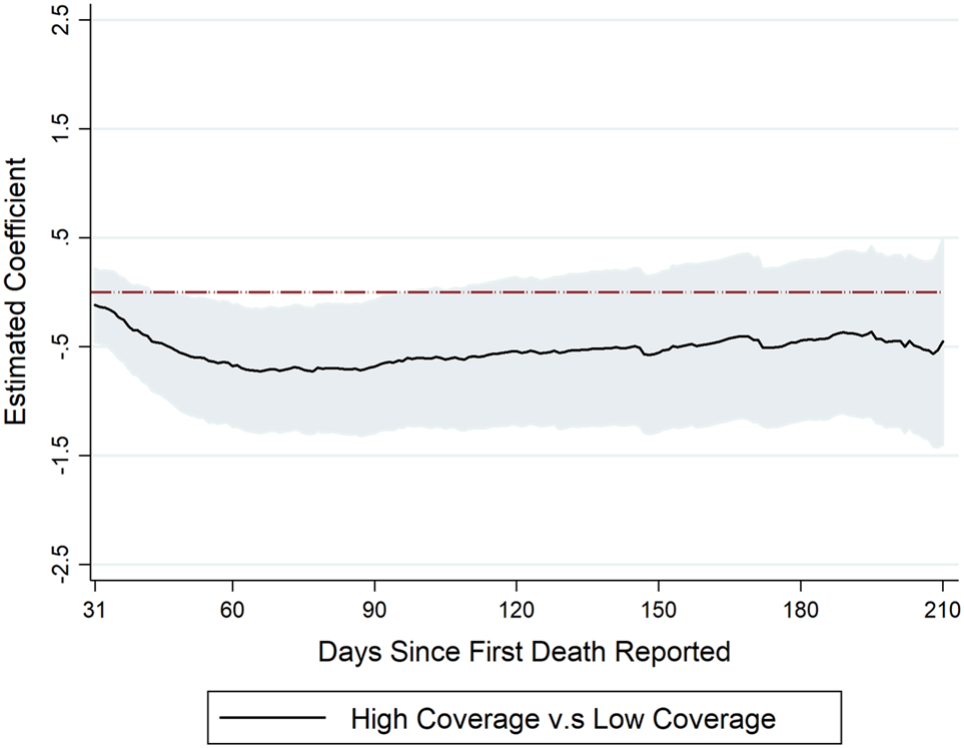
(Model 3) This figure summarizes the estimated effects of high BCG coverage (vs low BCG coverage) on cumulative deaths in logarithm since the first death reported. The coefficients are estimated effects of BCG vaccination program for day 31 to day 210, compared to the first 30 days. The effect of high BCG coverage estimates of $\alpha_{k}$ are from Eq. (3).

We summarize our findings as follows. See Table 1 and Figs. 1, 2 and 3 for an overview. The complete analysis results are in the Supplementary Material.

**Table 1:** Summary of effects of BCG in all three regression models: number of deaths on days 31–90, days 91–150 and days 151–210 compared with days 0–30.

	Dependent variable = log(# of total deaths)		
	days 0–30 vs days 31–90	days 0–30 vs days 91–150	days 0–30 vs days 151–210
**Model 1**			
All BCG*I (period of days)	− 0.870 (0.685)	− 0.263 (0.722)	0.055 (0.598)
Had BCG*I (period of days)	0.069 (0.736)	− 0.059 (0.780)	− 0.293 (0.674)
Marginal effect of all BCG (% reduction in outcome)	–	–	–
Number of observations	29,951		
**Model 2**			
Under 50*I (period of days)	− 1.266*** (0.461)	− 0.257 (0.530)	0.258 (0.531)
Over 50*I (period of days)	0.509 (0.347)	− 0.224 (0.467)	− 0.486 (0.549)
Marginal effect of under 50 (% reduction in outcome)	− 62.4%	–	–
Number of observations	18,601		
**Model 3**			
BCG high Coverage*I (period of days)	− 0.575** (0.251)	− 0.569 (0.333)	− 0.455 (0.370)
Marginal effect (% reduction in outcome)	− 43.8%	–	–
Number of observations	23,354		

***p < 0.01, **p < 0.05. Standard errors are clustered at country level. We calculate the marginal effects for the significant regression coefficients. In model 2, we convert the regression coefficients into marginal effects by exp($\alpha_{k}$ × mean of $Under50_{i}$) − 1. In model 3, we convert the regression coefficients into marginal effects by exp($\alpha_{k}$) − 1.

### Model 1

We first present the results of Model 1 that summarize the average effect of BCG vaccination program on the number of total deaths. Our dataset consists of summaries from 150 countries after filter for sample size and quality (see “Methods” section). Among these, 122 countries currently have BCG vaccination for the population, 20 countries who used to have BCG but do not currently have a national vaccination program, and 8 countries had never mandated BCG vaccination. Figure 1 plots the estimated effects associated with BCG programs in Model 1. We find countries with a current BCG vaccination program are associated with a lower cumulative number of deaths for the second and the third month since the first reported death, while the effects are not statistically significant (Fig. 1 left). No effects are found for countries that only had BCG vaccination in the past (Fig. 1 right). We summarize the average effect of BCG by comparing the growth in deaths between the first month and subsequent periods grouped by every 60 days in Table 1. The estimates suggest that countries with a current national BCG vaccination tend to have lower numbers of deaths, although the coefficients are not statistically significant. For countries with a past BCG program, the results again fail to show a significant link between BCG vaccination and both outcomes. The analysis also suggests that the effect of a BCG program might depend on the vaccinated cohort/age groups. For example, BCG vaccinations may have different levels of protective effects on seniors versus young adults. To further test this hypothesis, we next turn to examine the effect of BCG by linking the BCG coverage rate for different age groups and the COVID-19 outbreak.

### Model 2

We introduce Model 2 to further examine the effect of protection by past vaccination. A surprising finding is that BCG shows a stronger protection during days 31–60 since the first death was reported relative to days 0–30 if a country has higher coverage for the population under 50 years old. As shown in Fig. 2, the higher BCG coverage for the population under 50-year-old has significantly less deaths between day 31 and day 60 since the first death was reported (Fig. 2 left). However, protective effects of population coverage for those older than 50 years old are not statistically significant (Fig. 2 right). The result suggests that a country may have fewer deaths if its younger population is vaccinated. Yet a higher BCG coverage of the older population did not exhibit a similar effect.

In Table 1, we summarize the relationship between BCG vaccine and COVID-19 by comparing the numbers of deaths during days 0–30, days 31–90, days 91–150, and days 151 and beyond. We find that relative to days 0–30, for a country with the average BCG coverage level among all countries for under-50-year-olds (76.6%), the number of deaths is 62.4% lower than those without BCG coverage during days 31–60. This is the most statistically significant result of our analysis and remains significant when we exclude low-income countries or use an alternative isolation policy index in the model (see Supplementary Table 7).

### Model 3

Model 3 analyzes how BCG coverage as a proportion protects against COVID-19. We find borderline significant effects of BCG coverage as shown in Fig. 3: higher BCG coverage seems to help slow down the growth in both COVID-19 related deaths. Specifically, compared to countries with low BCG coverage (BCG coverage rate falls into the 1st quintile), countries with high BCG coverage (BCG coverage rate falls into the second quintile and above) start to exhibit a significantly slower growth rate for deaths immediately after the first month since the first death reported, though the protection is not statistically significant. However, almost starting from the 60th day after the first death confirmed, we observe a strong and significant protection on COVID-19, which lasts for one month (until day 90). After that, the impact gradually turns to be weak and disappears. To further examine to what extent the impact of high BCG coverage rate is, we again take the first month since first death as the reference period and group the rest days into three periods: days 31–90, days 91–150 and days 151–210. As shown in Table 1, our estimates suggest that high BCG coverage remarkably reduces the COVID-19 related death by 43.8% in the first period. The impact is no longer significant in the second and third period. We also perform analysis leveraging a continuous BCG coverage metric in Supplementary Table 8.

### Robustness

In all models, we find that the effect of BCG is stronger on the number of deaths during the early stage of the pandemic. Furthermore, we explore the robustness of our results by re-estimating all the three models with an alternative time-varying policy stringency covariate and different estimation samples. First, we replace the government response index with the policy stringency index. Second, we re-run the models in samples with different specifications. The results are all shown in Supplementary Tables 6–8. In sum, we find robust evidence suggesting that the BCG vaccine provides a protective effect against the COVID-19 outbreak at the beginning period, especially for countries with a higher BCG coverage rate, but limited effect later on.

### Heterogeneous effects of BCG

After observing the dynamics in BCG effects, we further estimate Eq. (5) to shed light on heterogeneity in the effect of BCG. Table 2 summarizes the coefficients of triple interaction terms between BCG indicators, time periods grouped by 60 days, and government response index (e.g., $\lambda_{k}$ and $\theta_{k}$ in Eq. 5). In Model 1, we find positive and significant coefficients, suggesting that a higher government response index offsets the effect of BCG on mortality for all time intervals. We also observe similar attenuating effects of government response index in Model 2, where all interaction terms with Under50 show positive and significant coefficients. We find similar estimates on the triple-interaction terms in Model 3, which only includes countries that have ever administered BCG vaccinations. Full estimates of Table 2 are available in Supplementary Tables 9–11. Results are similar when we replace the government response index with the policy stringency index in all interaction terms (Supplementary Tables 9–11). In summary, our results imply that the BCG protection diminishes when government intervention kicks in, which is consistent with the dynamic BCG vaccination effects we found.

**Table 2:** Summary of effects of BCG interacted with government response index.

	Dependent variable = log(# of total deaths)		
	days 0–30 vs days 31–90	days 0–30 vs days 91–150	days 0–30 vs days 151–210
**Model 1**			
All BCG*I (period of days)*Gov’t Index	0.050** (0.022)	0.042** (0.018)	0.085*** (0.020)
Had BCG*I (period of days)*Gov’t Index	0.008 (0.026)	− 0.005 (0.021)	0.040 (0.025)
Number of observations	29,951		
**Model 2**			
Under 50*I (period of days)*Gov’t Index	0.054*** (0.019)	0.082*** (0.020)	0.084*** (0.023)
Over 50*I (period of days)*Gov’t Index	− 0.012 (0.020)	− 0.031 (0.023)	− 0.029 (0.033)
Number of observations	18,601		
**Model 3**			
BCG High Coverage*I (period of days)*Gov’t Index	0.045*** (0.017)	0.037 (0.022)	0.040 (0.030)
Number of observations	23,354		

***p < 0.01, **p < 0.05. Standard errors are clustered at country level.

## Discussion

From the earliest history of BCG vaccination as a prevention for tuberculosis, it has been observed that BCG elicits a variety of effects that impact the immune system beyond conferring specific immunity to *Mycobacterium tuberculosis*^31,32^. While the primary mechanism of action of BCG, a mycobacterium, was originally thought to be via cross reactivity, it has also been observed that BCG has a broader effect^33^, that of “priming”, or what has recently been called “training” of the innate immune response^12,19^. This process stimulates the non-specific innate immune pathways to target other infections and even solid tumors in some cases^34^. Immunologists have long known about so called adjuvant effects of many bacterial species, including BCG, and have taken broad advantage of this phenomenon to enhance innate immune responses in a variety of settings including inclusion in autologous tumor vaccines^35^. However, the recent observations that this immune modulating effect can be long lasting, via trained immunity, provides an interesting mechanism that may explain some of the observations described here and may form a basis for potential COVID-19 protection in those individuals vaccinated with BCG, even years ago^36^. This observation motivates recent studies on the protectiveness of BCG vaccination against COVID-19. Yet the evidence is not conclusive.

Our results reconcile the existing different opinions towards the protection of BCG against COVID-19. We show that the protectiveness of BCG vaccination against COVID-19 is strongest during the very early stage of the pandemic, while as the government begins to implement social distancing policy, and other actions, the effect of BCG vaccination gradually disappears in the later stages. Although the old vaccines may protect from the virus, the self-protection actions as well as compliance to social distancing policies are still the most protective measures in absence of an effective COVID-19 vaccine.

Our study differs from previous studies in several aspects. A major difference is that previous studies do not fully address unobserved confounding factors, which may lead to a false sense of protection of BCG, even after carefully filtering out seemingly inhomogeneous countries based on human development index^19^, variability of test rates^28^, country population size^21^, income (socioeconomic wellbeing and level of development) of countries^8^, levels of social isolation policy^37^ and most importantly, the timing of the outbreak in each country^38^. We draw on models involving country and day fixed effect to account for country heterogeneities.

Secondly, analyses using mortality data at one specific time point during the pandemic render an aggregate effect in a specific time frame, while it is possible that the protection effect, if exists, varies across different stages of the pandemic. Our time-varying estimates of BCG protectiveness provide sufficient insights into dynamic interactions between BCG vaccination and public health policies. As the results showed, the protection effect is stronger at the beginning of the pandemic, suggesting that, without sufficient government interventions or public awareness, the BCG vaccine yields a stronger protection.

Our analysis focuses on COVID-19 mortality, since outcomes like reported cases are not as reliable as reported mortality. As suggested in literature, the number of confirmed cases could suffer more from underreporting^19,39^. Several factors, such as medical infrastructure, testing capacity, number of asymptomatic cases and criteria for testing could lead to the undertesting and underreporting of cases^19,28,40,41^. Therefore, mortality may serve as a better indicator for COVID-19 spread^41^, when high-quality cases data are not available. Future studies may incorporate corrected death or cases based on advanced projection models^42,43^.

Although the country fixed effect in our models can net out any country-level time-invariant factors such as medical infrastructure and healthcare access at the initial stage of the pandemic^41^, testing ability and medical resource availability may evolve during the pandemic thus affecting our estimations. Government response index may partially capture the trajectory of medical resource changes, but a better measure would be desired.

Another limitation of our analysis is that we did not take into account factors that will affect the rate of disease transmission, such as population density and traffic flow. As we observed, the severity of COVID-19 varies across urban and rural areas in a country. Since we rely on BCG program variations at country level, the population density variation within a country will be averaged out, thus possibly diluting the effect of BCG vaccine. If we can distinguish between samples in urban and rural areas and analyze them separately, then we might be able to investigate whether the effects of BCG vaccine vary across urban and rural areas. Comparing metropolitans in different countries (different BCG programs but similar population density and infrastructure, etc.) may shed light on the protective power of BCG against COVID-19 in an environment with a high volume of social activities and vice versa.

We recognize only clinical trials can conclusively confirm the protective effect of BCG vaccination. However, the current empirical evidence indeed sheds light on the benefit of a comprehensive vaccination program. It is intriguing that a targeted vaccination such as BCG may train our immune system to be protective against other diseases. Our study shows the protective effect is most significant for individuals 50 years or younger, but less so for those older than 50. This provides new research directions for the perplexing phenomenon of many adverse outcomes in the younger low-risk population. When the next inevitable outbreak occurs, we may not have an old vaccination to find new life in that pandemic, but the lesson we learn says it is the integrated health system that shields us from the danger.

## Supplementary Information

Supplementary Figures.

Supplementary Information 1.

Supplementary Information 2.

## Data Availability

Data and scripts are available from the corresponding authors upon request.
