# Supplementary material for: Reconcile the debate over protective effects of BCG vaccine against COVID-19
Source: Sci Rep. 2021 Apr 16;11:8356. doi: 10.1038/s41598-021-87731-9 (PMC8052320; doi:10.1038/s41598-021-87731-9)
Supplement: Supplementary file 1 — Supplementary Figures. [file 41598_2021_87731_MOESM1_ESM.pdf]

Supplementary Figure 1 to Figure 11

1 Vaccine Maps

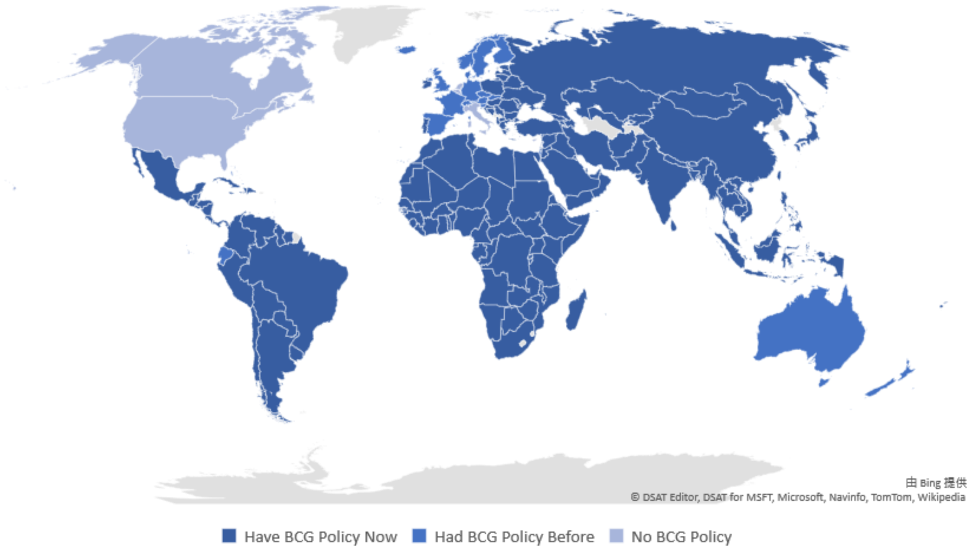

Supplementary Figure 1: BCG Policy Map

Notes: The figure is made using Microsoft Office 365 (version 2102, 64-bit) based on the data we obtain from BCG world atlas (www.bcgatlas.org) and other multiple complementary sources (see supplementary table 1 for more details about complementary sources).

(a) BCG Coverage under Age 50

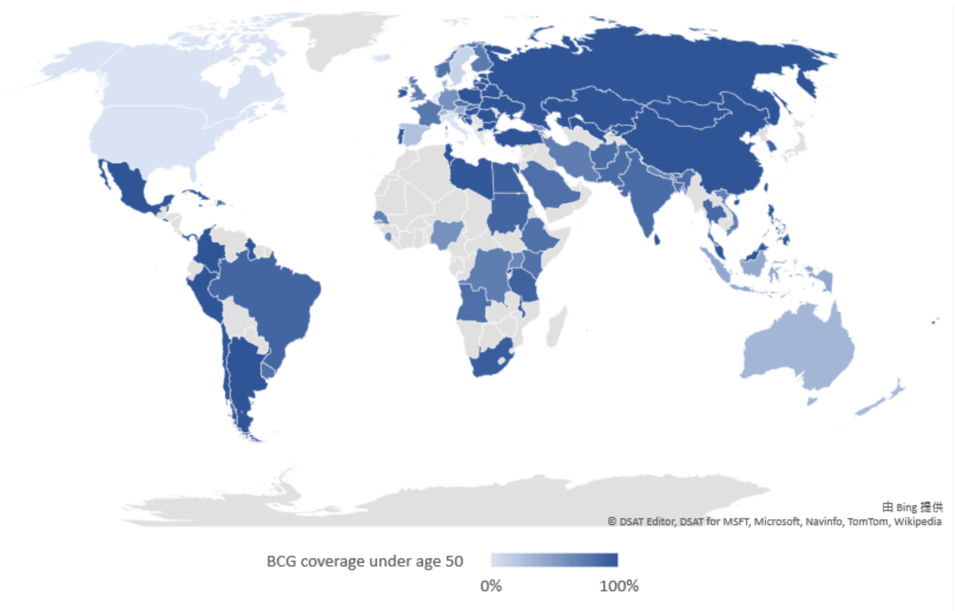

(b) BCG Coverage over Age 50

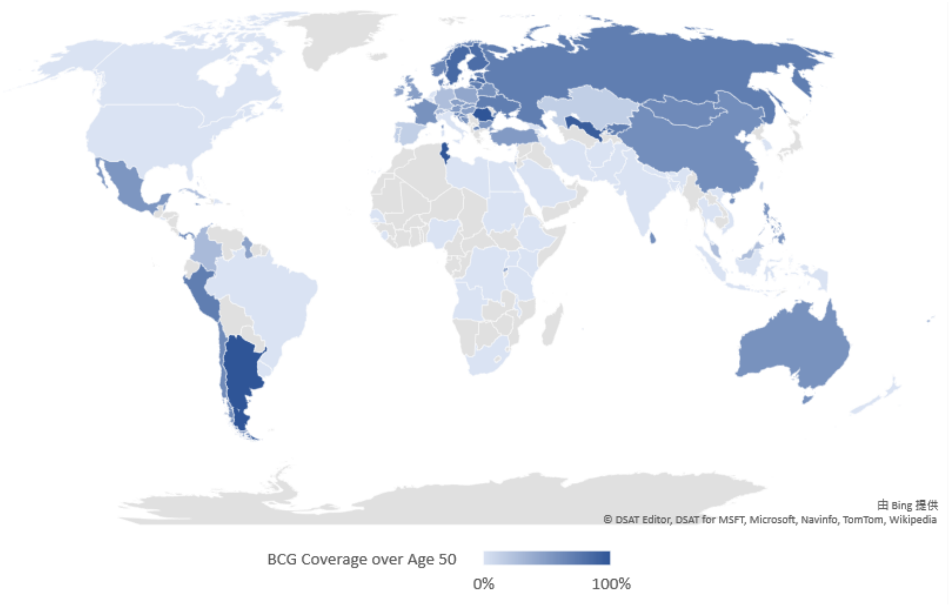

Supplementary Figure 2: BCG Coverage under and over Age 50 Map

Notes:(a)The BCG coverage under age 50 is estimated by the proportion of years between 1970 and 2019 with a national BCG vaccination program (b)The BCG coverage over age 50 is estimated by the proportion of years between 1935 and 1969 with a national BCG vaccination program.

The figure is made using Microsoft Office 365 (version 2102, 64-bit) based on the data we obtain from BCG world atlas (www.bcgatlas.org) and other multiple complementary sources (see supplementary table 1 for more details about complementary sources).

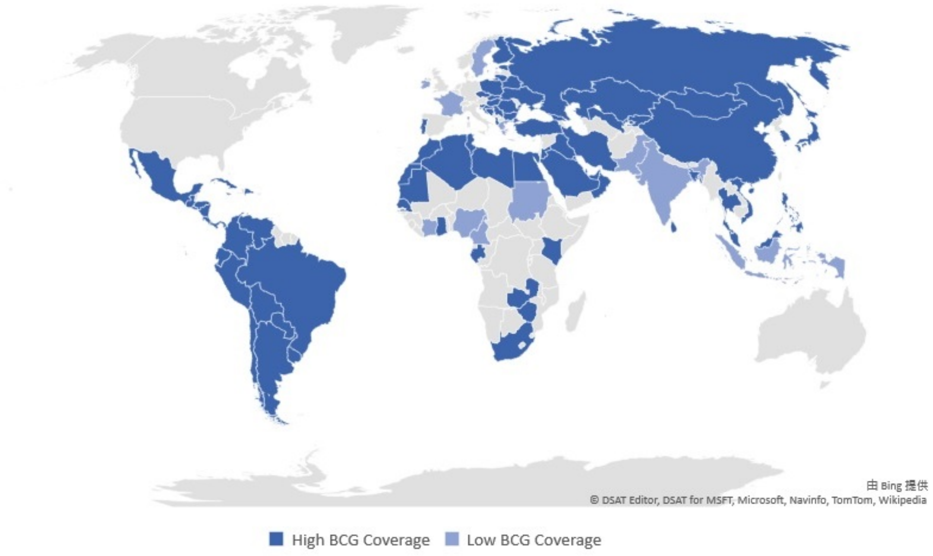

Supplementary Figure 3: BCG High and Low Coverage Map

Notes: A country is in high BCG coverage list if its average coverage of one-year-olds receiving one dose of BCG vaccine between 1990 and 2018 is higher than the first quintile (2nd quintile and above) across all countries.
The figure is made using Microsoft Office 365 (version 2102, 64-bit) based on the data we obtain from WHO Global Health observatory data repository (https://apps.who.int/gho/data/node.home).

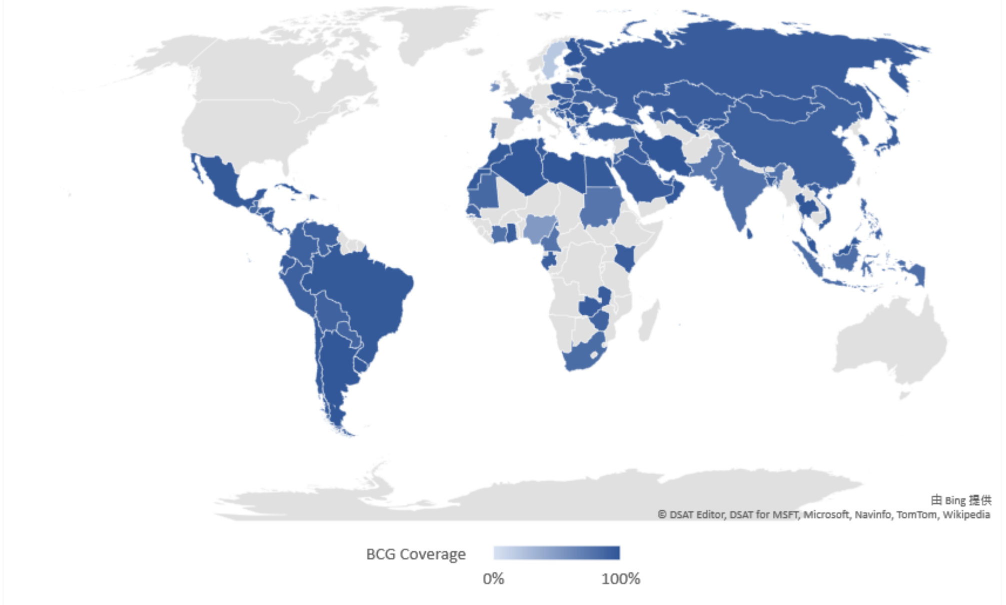

Supplementary Figure 4: BCG Coverage Map (average coverage from 1990 to 2018)

Notes: The figure is made using Microsoft Office 365 (version 2102, 64-bit) based on the data we obtain from WHO Global Health observatory data repository (https://apps.who.int/gho/data/node.home).

2 Distribution of BCG Vaccination Coverage

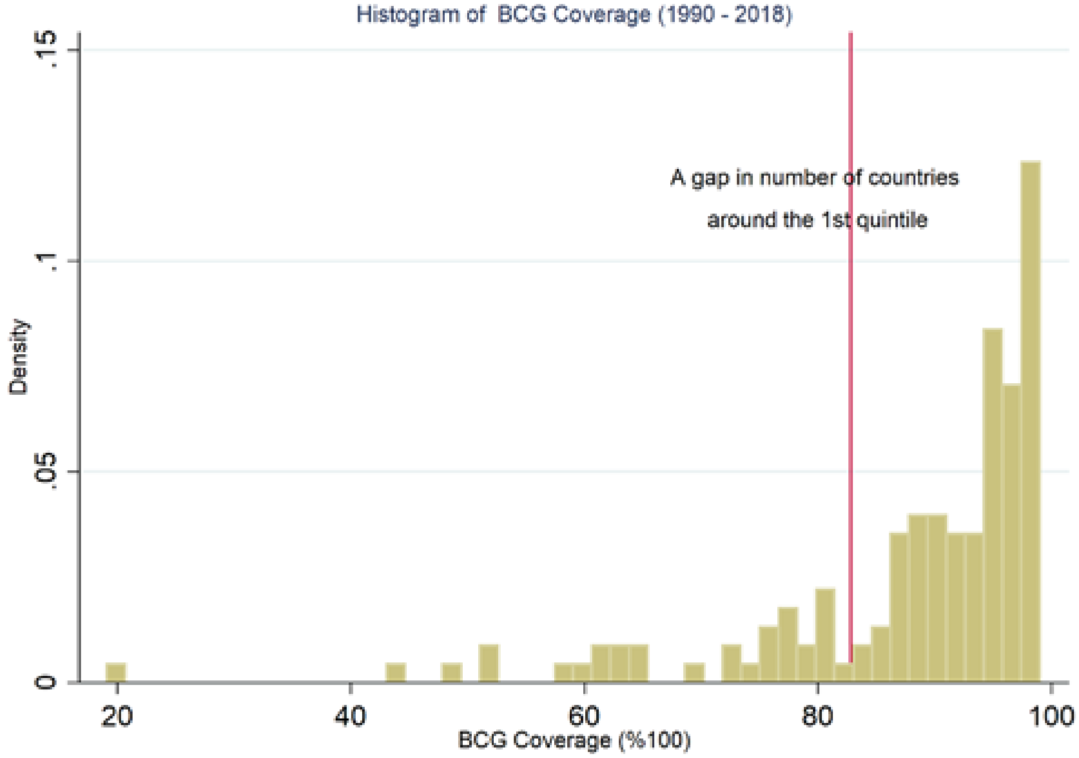

Supplementary Figure 5: Distribution of BCG Vaccination Coverage

3 Model 2 Using Different Age Cut-offs

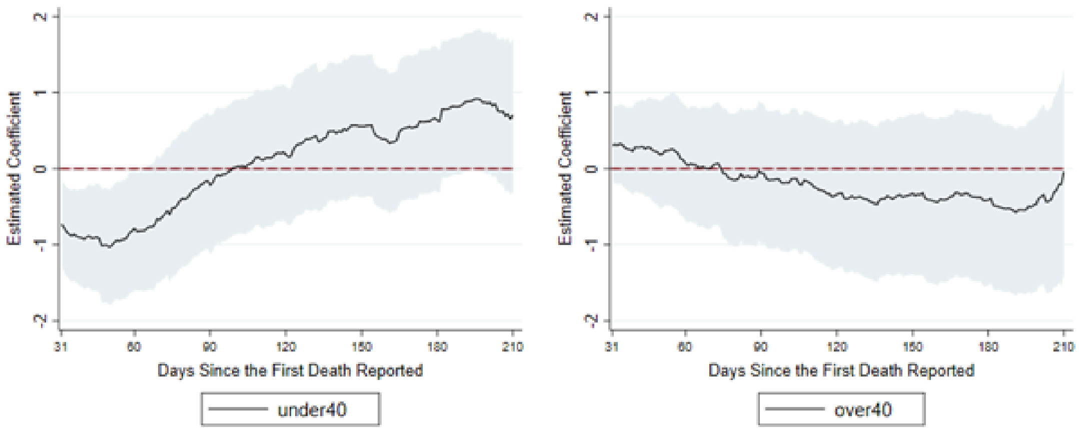

Supplementary Figure 6: Model 2, Age Cut-off at 40

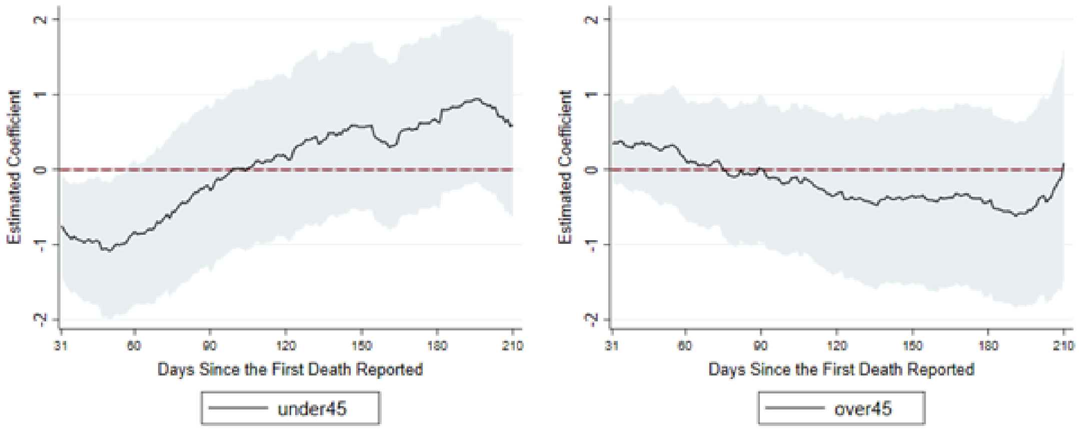

Supplementary Figure 7: Model 2, Age Cut-off at 45

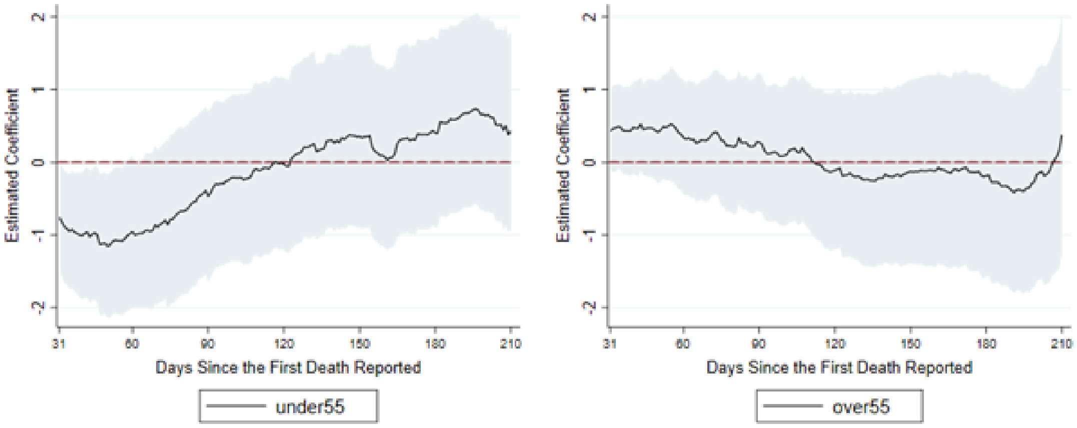

Supplementary Figure 8: Model 2, Age Cut-off at 55

4 Weekly Analysis in Primary Sample

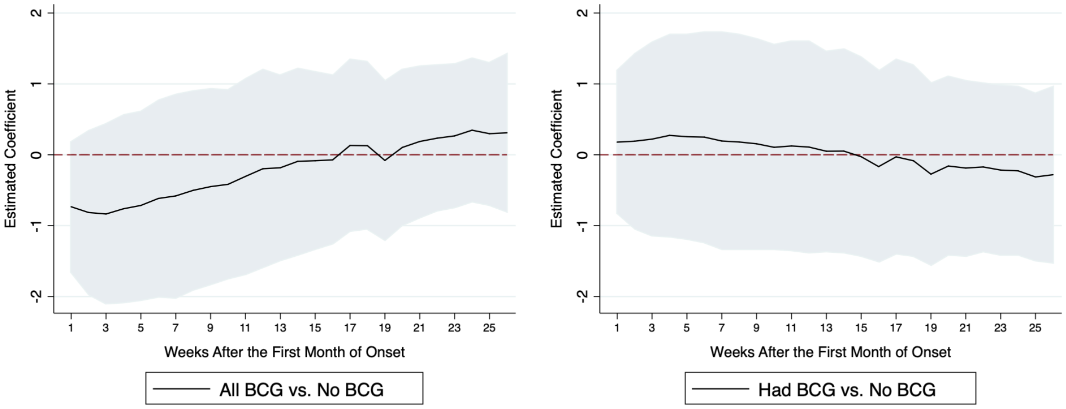

Supplementary Figure 9: Weekly Analysis Using Model 1

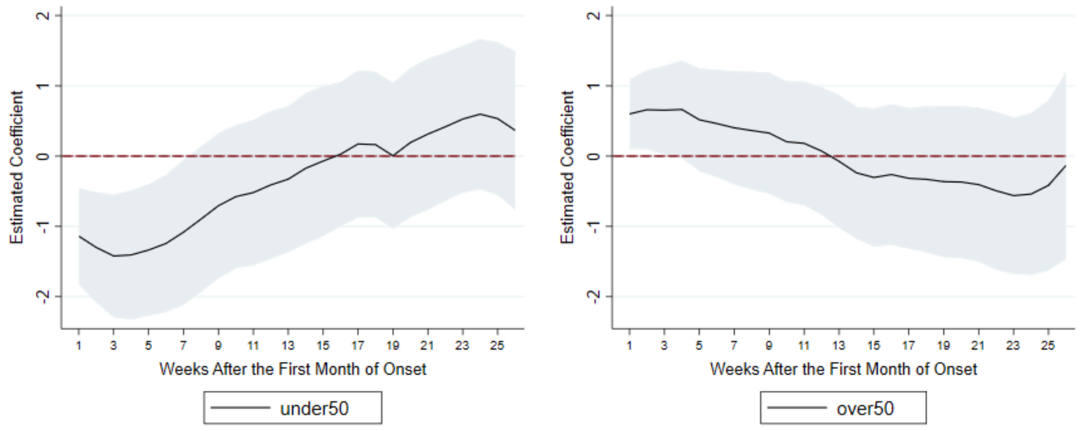

Supplementary Figure 10: Weekly Analysis Using Model 2

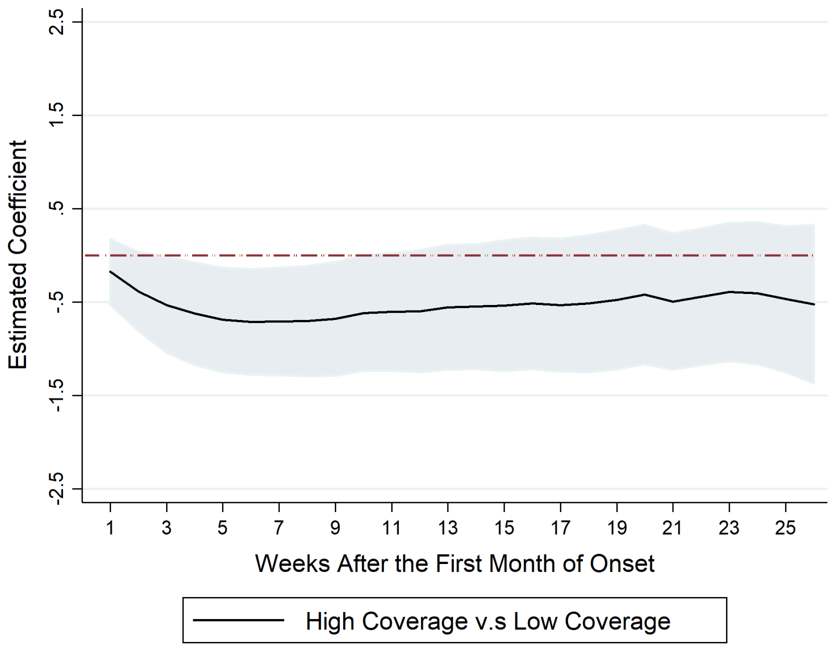

Supplementary Figure 11: Weekly Analysis Using Model 3
