# Supplementary material for: Reconcile the debate over protective effects of BCG vaccine against COVID-19
Source: Sci Rep. 2021 Apr 16;11:8356. doi: 10.1038/s41598-021-87731-9 (PMC8052320; doi:10.1038/s41598-021-87731-9)
Supplement: Supplementary file 3 — Supplementary Information 2. [file 41598_2021_87731_MOESM3_ESM.pdf]

Reconcile the Debate over Protective Effects of BCG Vaccine Against COVID-19

Wei Fu[1,2,^], Pei-Chuan Ho[3,^], Chia-Lun Liu[1,2,^], Kai-Teh Tzeng[3], Nawar Nayeem[3], Jonni S. Moore[2], Li-San Wang[1,2,*], Shin-Yi Chou[1,2,3,4,*]

[1] Penn Neurodegeneration Genomics Center, Perelman School of Medicine, University of Pennsylvania, Philadelphia PA, USA

[2] Department of Pathology and Laboratory Medicine, Perelman School of Medicine, University of Pennsylvania, Philadelphia PA, USA

[3] Department of Economics, Lehigh University, Bethlehem, PA, USA

[4] National Bureau of Economic Research, Cambridge, MA, USA

^ co-first author

** co-corresponding author*

Supplementary Table 1: BCG Policy by Country

Country	BCG Start Year	BCG Ended Year	Population 2020 (Million)	Income Level
**Panel A: Countries with BCG covered for all population currently**				
Afghanistan [30]	1978[1]		38.1	Low
Albania			2.9	Upper middle
Algeria			43.3	Upper middle
Argentina [12]	1935[3]		45.5	Upper middle
Azerbaijan			10.1	Upper middle
Bangladesh	1979[5]		169.8	Lower middle
Belarus	1950[4]		9.4	Upper middle
Benin			12.1	Low
Bolivia			11.5	Lower middle
Bosnia and Herzegovina	1950[5]		3.5	Upper middle
Brazil	1976[5]		213.9	Upper middle
Bulgaria	1951[5]		6.9	Upper middle
Burkina Faso			20.9	Low
Burma			54.8	Lower middle
Cameroon			26.0	Lower middle
Central African Republic			4.9	Low
Chad			16.3	Low
Chile	1948[5]		18.5	High
China [10]	1949[1]		1,432.7	Upper middle
Colombia	1960[5]		50.2	Upper middle
Congo (Brazzaville)			5.7	Lower middle
Congo (Kinshasa) [33]	1984[1]		89.5	Low
Costa Rica			5.0	Upper middle
Cote d'Ivoire			26.2	Lower middle

Table Supplementary 1 Continued: BCG Policy by Country

Country	BCG Start Year	BCG Ended Year	Population 2020 (Million)	Income Level
Croatia [41]	1948[5]		4.1	High
Cuba [35]	1962[1]		11.5	Upper middle
Dominican Republic [32]	1978[1]		11.1	Upper middle
Egypt [21]	1974[1]		102.9	Lower middle
El Salvador	1970[5]		6.5	Lower middle
Estonia	1945[4]		1.3	High
Ethiopia [42]	1980[1]		112.8	Low
Gabon			2.2	Upper middle
Georgia [28]	1991[3]		3.9	Upper middle
Ghana [33]			30.7	Lower middle
Greece [25]			11.1	High
Guatemala			17.9	Upper middle
Guinea [6]			13.8	Low
Honduras [17]			9.7	Lower middle
Hungary	1953[5]		9.6	High
India	1978[2]		1,383.2	Lower middle
Indonesia	1999[5]		272.2	Lower middle
Iran	1984[1]		83.6	Upper middle
Iraq [5]			41.5	Upper middle
Ireland	1955[5]		4.9	High
Jamaica [6]	1951[1]		2.9	Upper middle
Japan			126.5	High
Jordan [7]			10.2	Upper middle
Kazakhstan [13]	1965[2]		18.8	Upper middle
Kenya [26]	1980[2]		53.5	Lower middle

Table Supplementary 1 Continued: BCG Policy by Country

Country	BCG Start Year	BCG Ended Year	Population 2020 (Million)	Income Level
Korea, South	1975[5]		51.5	High
Kuwait [38]			4.3	High
Kyrgyzstan	1945[4]		6.3	Lower middle
Liberia			5.1	Low
Libya [4]	1971[1]		6.7	Upper middle
Lithuania [20]	1945[4]		2.9	High
Madagascar			27.7	Low
Malawi [34]	1974[3]		20.3	Low
Malaysia [27]	1961[3]		32.9	Upper middle
Mali			20.3	Low
Mauritania			4.8	Lower middle
Mauritius [44]	1951[1]		1.3	Upper middle
Mexico	1951[5]		133.9	Upper middle
Moldova			4.0	Lower middle
Mongolia	1948[5]		3.2	Lower middle
Morocco [45]			37.1	Lower middle
Mozambique			32.3	Low
Nepal [1]	1986[1]		30.3	Low
Nicaragua			6.4	Lower middle
Niger			24.1	Low
Nigeria	1991[5]		206.2	Lower middle
Oman			5.1	High
Pakistan [36]	1978[5]		208.4	Lower middle
Panama [11]	1949[4]		4.3	High
Paraguay			7.1	Upper middle

Table Supplementary 1 Continued: BCG Policy by Country

Country	BCG Start Year	BCG Ended Year	Population 2020 (Million)	Income Level
Peru	1945[5]		33.3	Upper middle
Philippines	1951[4]		109.7	Lower middle
Poland [39]	1955[1]		37.9	High
Portugal [2]	1965[1]		10.2	High
Qatar			2.8	High
Romania	1928[5]		19.4	Upper middle
Russia	1945[4]		143.8	Upper middle
Rwanda	1956[5]		13.1	Low
Saudi Arabia [37]	1979[1]		34.7	High
Senegal	1986[5]		17.2	Lower middle
Serbia			6.9	Upper middle
Sierra Leone	1990[5]		8.0	Low
Singapore [9]	1955[1]		5.9	High
Somalia [18]			16.1	Low
South Africa [16]	1973[1]		58.7	Upper middle
South Sudan [22]			13.6	Low
Sri Lanka [3]	1949[1]		21.1	Upper middle
Sudan	1976[5]		43.5	Lower middle
Syria			18.9	Low
Taiwan	1965[2]		23.8	High
Tanzania	1975[4]		62.8	Low
Thailand	1977[4]		69.4	Upper middle
Tunisia	1928[2]		11.9	Lower middle
Turkey	1952[5]		83.8	Upper middle
Uganda	1987[5]		47.2	Low

Table Supplementary 1 Continued: BCG Policy by Country

Country	BCG Start Year	BCG Ended Year	Population 2020 (Million)	Income Level
Ukraine	1945[4]		43.6	Lower middle
United Arab Emirates			9.8	High
Uruguay	1980[5]		3.5	High
Uzbekistan	1937[5]		33.2	Lower middle
Venezuela [40]			33.2	Upper middle
Vietnam	1985[4]		98.4	Lower middle
Yemen			30.2	Low
Zambia [31]			18.7	Lower middle
Zimbabwe [43]			17.7	Lower middle
**Panel B: Countries with BCG covered for all population in the past**				
Australia	1950[5]	1985	25.4	High
Austria	1952[2]	1990	8.8	High
Bahrain [19]		2003	1.7	High
Czechia	1953[4]	2010	10.6	High
Denmark	1946[5]	1986	5.8	High
Ecuador [23]	1986[1]		17.3	Upper middle
Finland	1941[5]	2006	5.6	High
France	1950[5]	2007	65.7	High
Germany	1961[5]	1998	82.5	High
Israel [15]	1955[5]	1982	8.7	High
New Zealand [14]	1976[5]	1990	4.8	High
Norway [8]	1947[4]	2009	5.4	High
Slovakia [24]	1953[1]	2012	5.5	High
Slovenia	1947[5]	2005	2.1	High
Spain	1965[5]	1981	46.5	High

Table Supplementary 1 Continued: BCG Policy by Country

Country	BCG Start Year	BCG Ended Year	Population 2020 (Million)	Income Level
Sweden	1940[5]	1975	10.1	High
Switzerland	1960[3]	1987	8.7	High
United Kingdom	1953[5]	2005	67.3	High
**Panel C: Countries without BCG covered for all population**				
Belgium			11.6	High
Canada	1943[4]		37.6	High
Cyprus [29]			1.2	High
Italy	1970[4]		59.1	High
Lebanon			6.0	Upper middle
Netherlands [45]		1979	17.2	High
Trinidad and Tobago		1976	1.4	High
United States			331.4	High

[1]Year when Mass BCG Immunization program started.
[2]Year when BCG covered for all newborn started.
[3]Year when BCG covered for some newborn started.
[4]Year of BCG Introduced obtained from Stefan Kirov's dataset (`https://github.com/kirovsa/covid19-bcg/blob/master/covid-bcg.pdf`) without detailed information about type of coverage.
[5]Year obtained from BCG World Atlas without detailed information (`http://www.bcgatlas.org/index.php`).

References

[1] National immunization programme and epi schedule of nepal. http://publichealthinnepal.blogspot.com/2016/06/national-immunization-programme-and-epi.html. Accessed: 06-06-2020.

[2] Portugal. http://venice.cineca.org/documents/portugal_ip.pdf. Accessed: 06-06-2020.

[3] News 1st. Is sri lanka's decades old bcg vaccine fighting off covid-19? https://tinyurl.com/y8qphyc7. Accessed: 06-06-2020.

[4] Suleiman Abusrewil. vaccination contribution to the sustainability and efficiency of health care systems in libya. https://www.fondation-merieux.org/wp-content/uploads/2017/01/vaccine-benefits-2017-suleiman-abusrewil.pdf. Accessed: 06-06-2020.

[5] Salih Alsariah and Taha Al-Azzawi. Observational study of clinical management of and outcome of bacille calmette-guÉrin vaccine adverse reactions: Baghdad/alrasafa respiratory center. *World Journal of Pharmaceutical and Life Sciences*, 5(8):91–98, 2019.

[6] Henrice Altink. 'fight tb with bcg': Mass vaccination campaigns in the british caribbean, 1951–6. *Medical history*, 58:475–97, 10 2014.

[7] A Batieha, K Abo-Khadhra, M Rawashdah, H Jaddou, M Sheyyab, K Abo-Romman, and A Daoud. The effectiveness of bcg vaccination: the jordanian experience. *Journal of tropical pediatrics*, 44(5):288–290, 1998.

[8] Jacob Berild and Trude Arnesen. Tuberculosis vaccine (bcg vaccine) - health care counselor. https://tinyurl.com/y7lbgdee. Accessed: 06-06-2020.

[9] Cynthia BE Chee and Lyn James. The singapore tuberculosis elimination programme: the first five years. *Bulletin of the World Health Organization*, 81:217–221, 2003.

[10] Z. R. Chen, X H Wei, and Z. Y. Zhu. Bcg in china. *Chinese medical journal*, 95 6:437–42, 1982.

[11] Teresina P de Pinzon. Bcg vaccination in the republic of panama. *American review of tuberculosis*, 67(4):522–525, 1953.

[12] CAB Direct. `https://tinyurl.com/yb83uz3n`. Accessed: 06-06-2020.

[13] Michael Favorov, Mohammad Ali, Aigul Tursunbayeva, Indira Aitmagambetova, Paul Kilgore, Shakhimurat Ismailov, and Terence Chorba. Comparative tuberculosis (tb) prevention effectiveness in children of bacillus calmette-guerin (bcg) vaccines from different sources, kazakhstan. *PloS one*, 7(3), 2012.

[14] Sherwood Gillian. Review of neonatal bcg immunisation services in new zealand in 2006. `https://tinyurl.com/yb83uz3n`. Accessed: 06-06-2020.

[15] Samuel Gross, Ernesto Kahan, Liora Vesterman, Ofra Havkin, and Alex Leventhal. Adverse reactions to accidental forearm injection of bacille calmette-guerin vaccine in schoolchildren: 12-month cohort follow-up. *Clinical infectious diseases*, 38(10):1495–1497, 2004.

[16] AC Hesseling, LF Johnson, H Jaspan, MF Cotton, A Whitelaw, HS Schaaf, PEM Fine, BS Eley, BJ Marais, J Nuttall, et al. Disseminated bacille calmette-guérin disease in hiv-infected south african infants. *Bulletin of the World Health Organization*, 87:505–511, 2009.

[17] Cara Bess Janusz, Carlos Castañeda-Orjuela, Ida Berenice Molina Aguilera, Ana Gabriela Felix Garcia, Lourdes Mendoza, Iris Yolanda Díaz, and Stephen C Resch. Examining the cost of delivering routine immunization in honduras. *Vaccine*, 33:A53–A59, 2015.

[18] Irving Kaplan. *The Area Handbook for Somalia.* US Government Printing Office, 1969.

[19] Safaa Khawaja, Jaleela Jawad, Nermin Saeed, Nashwa Fawzy, Moez Azam, Ebrahim Romaihi, Kubra Nasser, and Bassma Saffar. Tuberculosis trends in the kingdom of bahrain, twelve years experience with the implementation of selective bcg vaccination strategy. pages 8–16, 09 2017.

[20] J Korablioviene, M Mauricas, S Caplinskas, G Zagrebneviene, and P Korabliov. The historical experience and practice of fight against tuberculosis in country which is one of the high drug resistant-tuberculosis (dr-tb) burden countries in european union (eu). *Journal of preventive medicine and hygiene*, 59(4):E328, 2018.

[21] M Madkour, AS Khalifa, et al. A critical review of bcg vaccination programme in egypt. *Journal of Tropical Medicine and Hygiene*, 80(7):144–146, 1977.

[22] Adut C Malual, Yuko Jowi, Grace Irimu, and Bashir Admani. Missed opportunities for immunization among children attending a paediatric outpatient clinic at juba teaching hospital. *South Sudan Medical Journal*, 11(2):36–39, 2018.

[23] Pablo Martinez. Implementation of Vaccine Programs in Ecuador. *Reviews of Infectious Diseases*, 11:S507–S508, 05 1989.

[24] Elena Marušáková, Ján Bielik, Klára Frecerová, Eva Nevická, and Darina Sedláková. Cancelling obligatory mass vaccination of newborns against tb in slovakia: predicted development. *University Review*, 6(1):2–11, 2012.

[25] JB McDougall. Tuberculosis in greece: an experiment in the relief and rehabilitation of a country. *Bulletin of the World Health Organization*, 1(1):103, 1948.

[26] The Government of Kenya Ministry of Health. National policy guidelines on immunization 2013. `https://tinyurl.com/yd56zkq8`. Accessed: 06-06-2020.

[27] The Government of Malaysia Ministry of Health. Bcg revaccination. `www.moh.gov.my`. Accessed: 06-06-2020.

[28] Veriko Mirtskhulava, Russell Kempker, Katherine L Shields, Michael K Leonard, Tengiz Tsertsvadze, Carlos del Rio, Archil Salakaia, and Henry M Blumberg. Prevalence and risk factors for latent tuberculosis infection among health care workers in georgia. *The International Journal of Tuberculosis and Lung Disease*, 12(5):513–519, 2008.

[29] Carlotta Montagnani, Susanna Esposito, Luisa Galli, Elena Chiappini, Nicola Principi, and Maurizio de Martino. Recommendations for pediatric tuberculosis vaccination in italy. *Human Vaccines & Immunotherapeutics*, 12:00–00, 11 2015.

[30] Raveesha Mugali, Ghulam Mansoor, Sardar Parwiz, Najibullah Safi, Ariel Higgins-Steele, and Sherin Varkey. Improving immunization in afghanistan: results from a cross-sectional community-based survey to assess routine immunization coverage. *BMC Public Health*, 17, 04 2017.

[31] The Government of the Republic of Zambia. Financial sustainability plan for the immunization programme in zambia. `https://tinyurl.com/yddop4kt`. Accessed: 06-06-2020.

[32] Eddy Perez-Then, Gail Shor-Posner, Lee Crandall, and James Wilkinson. The relationship between nutritional and sociodemographic factors and the likelihood of children in the dominican republic having a bcg scar. *Revista panamericana de salud pública = Pan American journal of public health*, 21:365–72, 07 2007.

[33] Richard Phillips, Delphin Phanzu, Marcus Beissner, Kossi Badziklou, Elysée Luzolo, Fred Sarfo, Afiwa Halatoko, Yaw Amoako, Michael Frimpong, Abass Kabiru, Ebekalisai Piten, Issaka Maman, Bawimodom Bidjada, Adjaho Koba, Koffi Awoussi, Basile Kobara, Jörg Nitschke, Franz Wiedemann, Abiba Kere, and Karl-Heinz Herbinger. Effectiveness of routine bcg vaccination on buruli ulcer disease: A case-control study in the democratic republic of congo, ghana and togo. *PLoS neglected tropical diseases*, 9:e3457, 01 2015.

[34] JM Ponnighaus, E Msosa, PJK Gruer, NG Liomba, PEM Fine, JAC Sterne, RJ Wilson, L Bliss, PA Jenkins, and SB Lucas. Efficacy of bcg vaccine against leprosy and tuberculosis in northern malawi. *The Lancet*, 339(8794):636–639, 1992.

[35] Gail Reed and Miguel Galindo. Cuba's national immunization program. *MEDICC review*, 9:5–7, 10 2008.

[36] Erik Roelsgaard, Hans Christensen, and Erik Iversen. Bcg-vaccination programme in pakistan. *Bulletin of the World Health Organization*, 17(2):187, 1957.

[37] Fahad Saleh al Tayyeb. Bacillus calmett-guerin (bcg) vaccination "an overview from saudi arabia". *Journal of Antivirals & Antiretrovirals*, 8, 2016.

[38] MA Shaaban, M Abdul Ati, GM Bahr, JL Standford, DN Lockwood, and IC McManus. Revaccination with bcg: its effects on skin tests in kuwaiti senior school children. *European Respiratory Journal*, 3(2):187–191, 1990.

[39] I Szczuka. Adverse events following immunization with bcg vaccine in poland 1994-2000, 2002.

[40] Daniel Sánchez, Samir Sodha, Hannah Kurtis, Gladys Ghisays, Kathleen Wannemuehler, M. Danovaro-Holliday, and Alba Ropero Alvarez. Vaccination week in the americas, 2011: An opportunity to assess the routine vaccination program in the bolivarian republic of venezuela. *BMC Public Health*, 15, 04 2015.

[41] Goran Tešović. Childhood vaccinations in croatia. *Periodicum Biologorum*, 114:149–166, 2012.

[42] Asmamaw Ketemaw Tsehay, Getasew Tadesse Worku, and Yihun Mulugeta Alemu. Determinants of bcg vaccination coverage in ethiopia: a cross-sectional survey. *BMJ open*, 9(2):e023634, 2019.

[43] UNICEF. Factors influencing vaccine hesitancy and immunization coverage in zimbabwe: A rapid assessment. `https://tinyurl.com/yda4d2sq`. Accessed: 06-06-2020.

[44] AA Wagner et al. Bcg campaign in mauritius. *Tubercle*, 36(1):26–30, 1955.

[45] Wikipedia. Bcg vaccine. `https://en.wikipedia.org/wiki/BCG_vaccine`. Accessed: 06-06-2020.
